# Discovery of Novel Isoforms of Huntingtin Reveals a New Hominid-Specific Exon

**DOI:** 10.1371/journal.pone.0127687

**Published:** 2015-05-26

**Authors:** Albert Ruzo, Ismail Ismailoglu, Melissa Popowski, Tomomi Haremaki, Gist F. Croft, Alessia Deglincerti, Ali H. Brivanlou

**Affiliations:** Laboratory of Molecular Embryology, The Rockefeller University, New York, New York, United States of America; Institut Curie, FRANCE

## Abstract

Huntington’s disease (HD) is a devastating neurological disorder that is caused by an expansion of the poly-Q tract in exon 1 of the Huntingtin gene (HTT). HTT is an evolutionarily conserved and ubiquitously expressed protein that has been linked to a variety of functions including transcriptional regulation, mitochondrial function, and vesicle transport. This large protein has numerous caspase and calpain cleavage sites and can be decorated with several post-translational modifications such as phosphorylations, acetylations, sumoylations, and palmitoylations. However, the exact function of HTT and the role played by its modifications in the cell are still not well understood. Scrutiny of HTT function has been focused on a single, full length mRNA. In this study, we report the discovery of 5 novel *HTT* mRNA splice isoforms that are expressed in normal and *HTT*-expanded human embryonic stem cell (hESC) lines as well as in cortical neurons differentiated from hESCs. Interestingly, none of the novel isoforms generates a truncated protein. Instead, 4 of the 5 new isoforms specifically eliminate domains and modifications to generate smaller HTT proteins. The fifth novel isoform incorporates a previously unreported additional exon, dubbed 41b, which is hominid-specific and introduces a potential phosphorylation site in the protein. The discovery of this hominid-specific isoform may shed light on human-specific pathogenic mechanisms of HTT, which could not be investigated with current mouse models of the disease.

## Introduction

The *HTT* gene is evolutionarily conserved from arthropods to humans. The human *HTT* genomic locus on chromosome 4 (4p16.3) consists of 67 exons transcribed to an mRNA of 13481 bps (referred hereon as the canonical HTT isoform), encoding a protein of 3144 amino acids (aa), according to public genome annotations. HTT is expressed maternally in the fertilized egg and subsequently in all cells of the adult[1,2]. Mutations in the *HTT* locus have devastating consequences. Expansion of N-terminal polyQ repeats is sufficient to cause Huntington’s disease (HD), a lethal neurodegenerative disorder. In addition, homozygous deletion of *Htt* leads to embryonic lethality in the mouse, demonstrating that Htt function is necessary for early embryonic development.

*Htt* ^*—/-*^ mouse embryos die at E7.5 with severe defects in gastrulation and primitive streak patterning[3,4], thought to be due to primary effects on the visceral endoderm[5]. Work utilizing mice to model HD has been hampered by the inability of the mouse model to completely recapitulate human disease phenotypes[6,7]. This may be due to the temporal differences between mouse and human or to fundamental differences between the two species. Despite the fact that the *HTT* gene was identified more than 20 years ago and among the first human genes shown to be causal to a disease in a heterozygous background, the exact functions of the HTT protein remain unknown[8].

Investigation of the roles played in cells by HTT has mostly focused on the protein derived from the canonical mRNA that includes all 67 exons as well as cleavage fragments that occur during disease progression. In addition to the canonical mRNA, some studies have reported three shorter HTT isoforms: an alternatively spliced mRNA that eliminates exons 34 to 44 (originally named isoform B, hereafter referred to as *HTT-Δ34–44*) and one isoform that eliminates exon 28 (hereafter referred to as *Htt- Δ 28)* have both been reported in human adult brains[9,10], while a spliced form of HTT mRNA where intron 1 is not spliced out (called Exon1-Intron1 isoform) has been reported in patients with HD[11]. This last splice form produces a truncated protein limited to exon1, which has increased toxicity[11]. While the functions of these shorter isoforms are currently unknown, they have provided the first hint that the large *HTT* genomic locus can give rise to differentially spliced mRNAs potentially encoding different HTT proteins.

In order to decipher the function of *HTT* in humans, we began by investigating the presence and diversity of *HTT* transcripts in pluripotent human embryonic stem cells (hESCs). The canonical HTT isoform is expressed in both pluripotent mESCs and hESCs[5,12]. In this study, we utilize high-throughput RNA sequencing (RNA-seq) to scan the transcriptome of hESCs, both wild-type and HD mutants, to assess the presence of differentially spliced *HTT* mRNA transcripts. We report the discovery of five novel isoforms of *HTT* that can give rise to HTT protein variants lacking specific domains. We also identify, for the first time, a hominid-specific isoform of *HTT*. This splice isoform, due to its conservation only to great apes, could be an important factor in HD pathogenesis and might shed light on differences between mouse models and human phenotypes of HD. Our results identify previously unrecognized *HTT* mRNAs that encode different subtypes of HTT proteins and highlight the importance of studying all isoforms to completely understand HTT physiological and pathological functions.

## Results and Discussion

### RNA-seq analysis of normal and HD hESCs identifies new HTT splice variants

**Fig 1:**
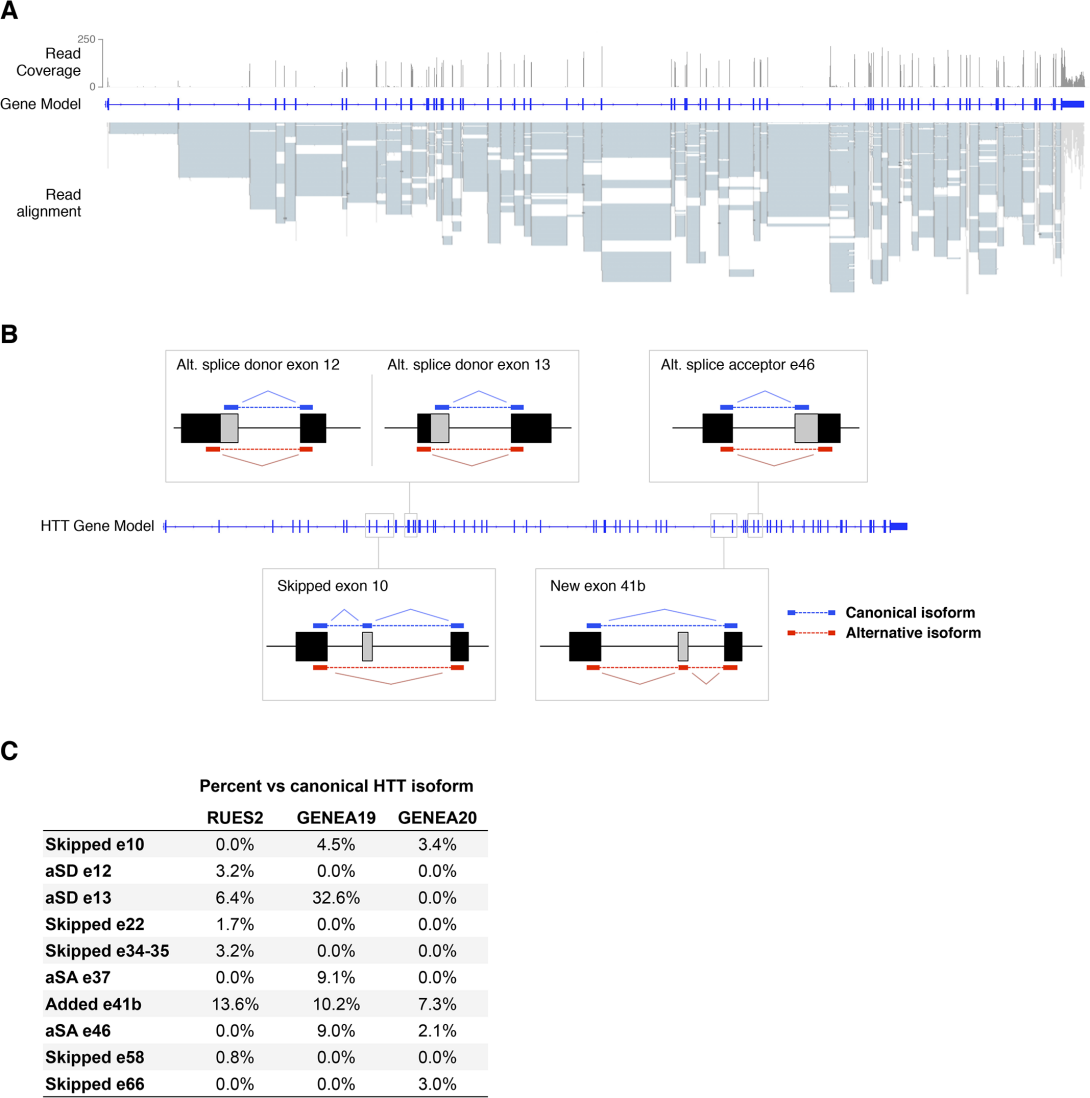
RNA-seq analysis reveals novel isoforms of *HTT*. Using Tuxedo software, 10 putative *HTT* splice isoforms were detected in RNA-seq data. (A) RUES2 RNA-seq reads were aligned to the hg19 genome, demonstrating good coverage of HTT mRNA used for the isoform analysis. (B) Diagram depicting the 5 HTT isoforms validated in this study. (C) Frequency of all detected isoforms across the three RNA-seq samples. aSA: alternative splice acceptor, aSD: alternative splice donor.

In order to detect *HTT* transcripts, RNA-seq was performed in 3 independent hESCs lines cultured under pluripotency conditions. The lines included RUES2, a female (XX) line originally derived in our laboratory[13,14] (NIHhESC-09-0013), and two hESC lines derived from sibling female (XX) embryos, one wild-type and one containing mutant *HTT* (Genea019 and Genea020, respectively[15]). Examination of *HTT* transcripts confirmed the expression of the canonical *HTT* mRNA in all three lines, with enough read coverage to perform isoform analysis (a range of 12,000–14,000 100-bp reads mapping the HTT locus—Fig 1A). RNA-seq demonstrated that RUES2 has a normal CAG repeat length, with one allele harboring 22, and the other one 24 CAGs (22/24). In agreement with previous characterization[15], Genea019 displayed normal CAG repeats (15/18), while Genea020 presented an extended CAG tract (17/48) that carries the signature for HD. Interestingly, we did not detect *HTT-Δ34–44*[9], *HTT-Δ28*[10], or *HTT-exon1-intron1*[11] mRNAs. The absence of these previously reported *HTT* splice isoforms in either normal or diseased hESC lines suggests that they might represent temporally regulated isoforms that emerge after differentiation. Our RNA-seq approach did reveal the presence of 10 additional putative *HTT* splice variants, each with one or more exons spliced out from the canonical *HTT* isoform, as well as one longer isoform that incorporates a previously unreported exon (Fig 1B). Most of these new HTT transcripts were found at low levels (<5% reads compared to the canonical HTT isoform), and not always detected in all three samples, suggesting that these putative splice variants of HTT are indeed rare transcripts, potentially explaining why they have not been previously reported (Fig 1C). However, the longer variant (incorporating a new exon between exons 41 and 42) was consistently found to be relatively prevalent –about 7–13% of the major *HTT* isoform- among all samples analyzed.

### Validation of new HTT transcripts by qPCR

**Fig 2:**
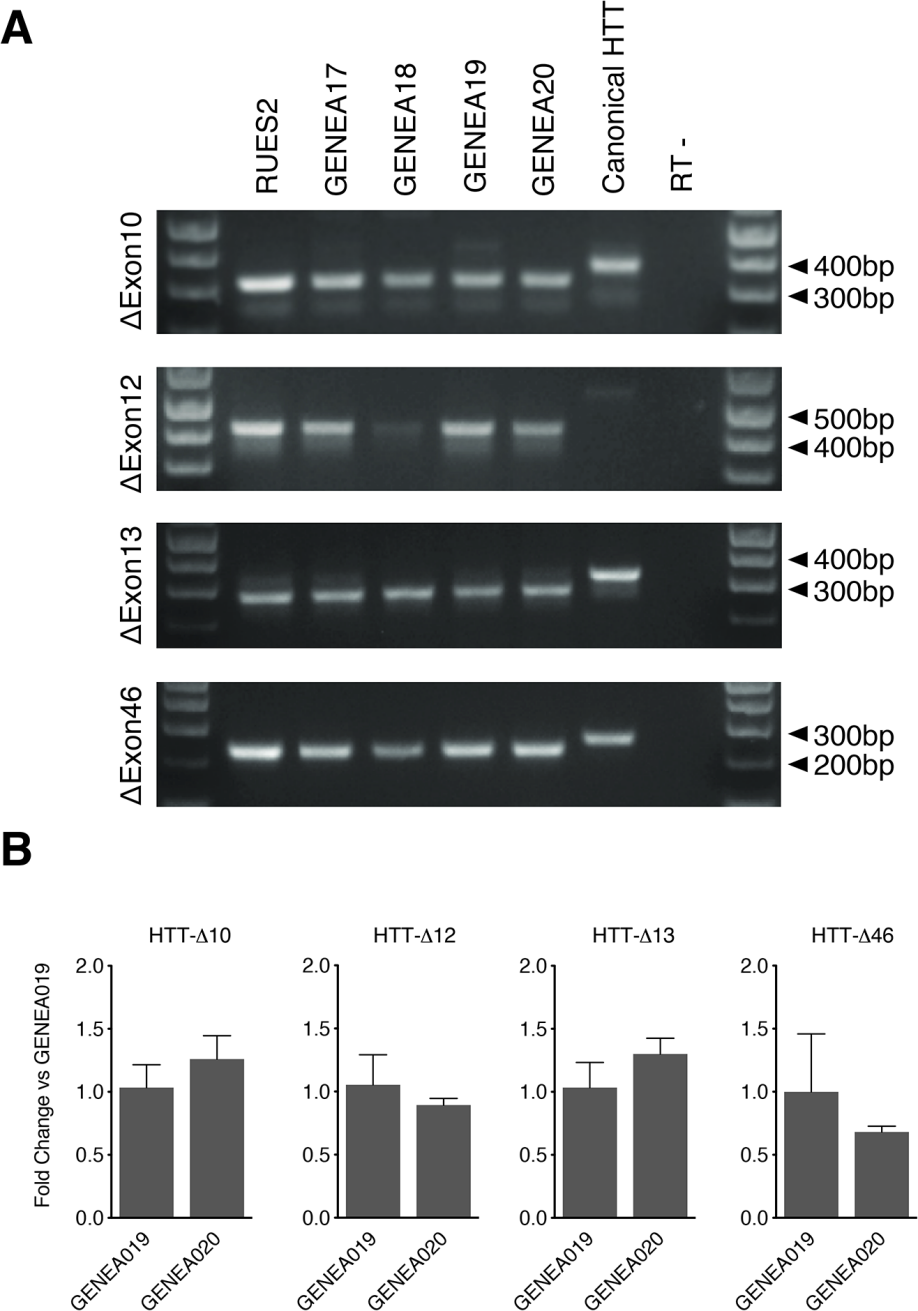
PCR and qPCR validation of novel HTT isoforms. (A) RT-PCR results with primers specific for each individual splice isoform, detecting expression of HTT-Δ10, Δ12, Δ13 and Δ46 in hESCs cells. A plasmid containing the canonical full-length HTT was used to control for non-specific amplification of canonical HTT mRNA. (B) Quantification of HTT isoform expression in hES cells through qPCR in GENEA019 and GENEA020 hESCs. Data represents mean + SEM of 3 replicates.

To validate the RNA-seq results, we used exon-specific PCR to independently assess the expression of the novel *HTT* isoforms. This analysis confirmed the presence of 5 of the 10 novel HTT transcripts detected by RNA-seq (Fig 2 and see below). The lack of detection of the other five might be due to artifacts from RNA-seq or to their very low abundance. We therefore focused on the 5 transcripts that were validated by PCR. Of these, 4 lacked an exon, either completely or partially: *HTT-Δ10*, *HTT-Δ12*, *HTT-Δ13* and *HTT-Δ46*, while the fifth, longer isoform incorporated the new exon: *HTT-41b*. Interestingly, these 5 novel *HTT* transcripts had two common attributes. First, they were all detected in both normal and HD hESC lines (Fig 2A and see below). To confirm that they were present in more than one HD line, we also examined the expression of the four shorter isoforms in two other, unrelated HD lines: a male (XY) Genea017 (12/40) and female (XX) Genea018 (17/46) (Fig 2A). All isoforms were expressed with no significant differences in expression levels between cell lines when assessed by qPCR (Fig 2B). The second attribute is that none of the splice isoforms is predicted to disrupt the HTT open reading frame (ORF), but they are instead predicted to encode proteins where specific internal domains are deleted or gained. The discovery of these 5 new transcripts brings the total number of HTT mRNA subtypes, including the full-length, to 9.

A number of post-translational modification sites have been detected in the 17 amino acid N-terminal portion (N17) of HTT, preceding the poly-Q repeat. In addition to phosphorylation and ubiquitination sites, this sequence has been shown to include a nuclear localization signal[16], an ER targeting signal[17,18] and a more general localization signal, which under stress conditions causes HTT to be released from the ER and accumulate in the nucleus[19]. A nuclear export signal has been found on the C-terminus of the protein, between aa 2397–2406[20]. However, the rest of the HTT protein remains largely unexplored. Isoform-specific changes in HTT post-translational modifications due to missing amino acids are detailed below.

### HTT splice isoform 1: HTT-Δ10

**Fig 3:**
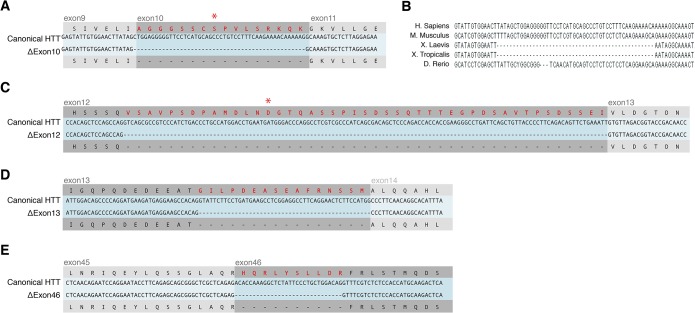
HTT protein consequences of the 4 novel shorter isoforms. (A, C-E) Alignment of sequencing data obtained from amplicons shown in Fig 2 to canonical HTT, confirming the isoform sequences obtained from the RNAseq analysis. Red protein sequences represent predicted lost amino acids. Amino acids highlighted with an asterisk represent Ser434 in (A) and Asp552 in (C), sites of phosphorylation and caspase cleavage, respectively. (B) Alignment of human, mouse, frog and zebrafish HTT mRNA sequences show that Exon10 is specifically missing in the gene model of frog Htt, while it is present in mammalians (human, mouse) and fish (zebrafish).

*HTT-Δ10* specifically lacks the 48bp-long exon 10, deleting amino acids 427 to 442 (Fig 3A). The splicing machinery connects exon 9 to 11 without disrupting the ORF, eliminating 16 aa from full length HTT. This domain of HTT has been previously shown to be the target of post-translational modifications. Cdk5 can phosphorylate Ser434, which is absent in the splice variant[21] (Fig 3A). This phosphorylation in turn decreases caspase-dependent cleavage of HTT[21]. Since shorter forms of HTT are typically associated with enhanced toxicity, the Ser434 post-translational modification could decreases toxicity, presumably indirectly, through its effects on proteolysis of HTT. Due to the absence of this site in the *HTT-Δ10* isoform, the protein predicted to be produced from this transcript might be more toxic than the full-length isoform.

Interestingly, this *HTT* splice variant is evolutionarily conserved and detected in amphibians. Examination of *Xenopus laevis* and *tropicalis* sequence shows that the region corresponding to Exon10 is absent in the canonical *Xenopus htt* transcripts (Fig 3B). Indeed, when we performed RNA-seq of *X*. *laevis* gastrula, we find that this isoform (*htt-Δ10)* is the most prevalent in the embryo, representing ~96% of the total *htt* transcripts (S1 Fig). However, we also identified transcripts that retained exon 10 (*htt-ex10i*), which accounted for ~4% of all *htt* transcripts. These results were confirmed by RT-PCR (data not shown). Because of the stark contrast between human and *Xenopus* in the prevalence of transcripts with or without exon 10, we further investigated the temporal and spatial expression of these two isoforms. We found that while the major *Xenopus* isoform *htt-Δ10* is expressed at all stages analyzed during early development (S1 Fig), the other isoform (*htt-ex10i*) is dramatically upregulated at stage 28, around the time when the nervous system is fully developed. Dissection of tadpoles at stage 35 into four regions showed that, consistent with expression in the CNS, the *htt-ex10i* form is predominantly expressed in the head and dorsal regions (S1 Fig). Thus, these results suggest a possible link between development of the nervous system and expression of the *htt-ex10i* isoform, which was confirmed by analysis of *HTT-Δ10 and HTT-ex10i* expression levels in hESCs differentiating towards the neuronal lineage (see below).

### HTT splice isoform 2: HTT-Δ12

*HTT-Δ12* isoform is generated by using an alternative splice donor site at nucleotide 1760, leading to a predicted protein that lacks 135 nucleotides from the 3’ end of Exon 12. This eliminates 45 amino acids from the canonical HTT protein, amino acids 539 to 583 (Fig 3C). Interestingly, this splice form also eliminates another caspase cleavage site at D552 that is used to cleave both the normal as well as the CAG-extended mutant HTT[22]. N-terminal fragments of full length HTT protein generated by caspase cleavage at D552 have been detected in the brains of controls and HD patients as well as HD mouse models and controls[22]. In human brains, increased concentration of N-terminal fragments in cortical neurons are a hallmark of HD[8]. Mouse studies have shown that HTT fragments appear before the onset of neurodegeneration[23]. N-terminal fragments of HTT translocate into the nucleus[24] and are considered to be more toxic than the full-length form[25]. Therefore, unlike *HTT-Δ10*, *HTT-Δ12* is expected to be resistant to caspase cleavage and thus a less toxic isoform.

### HTT splice isoform 3: HTT-Δ13

*HTT-Δ13* isoform lacks 16 amino acids (609 to 624) at the C-terminus of Exon 13 (Fig 3D). The mRNA is spliced in-frame and is thus expected to produce a near full-length HTT protein. No known post-translational modification sites are included in the missing sequence. However, like *HTT-Δ10* and *HTT-Δ12*, *HTT-Δ13* affects the predicted armadillo-like repeat section of HTT and thus might affect HTT structure or protein-protein interactions.

### HTT splice isoform 4: HTT-Δ46

*HTT-Δ46* isoform, missing 10 amino acids at the N-terminal side of exon 46, is also predicted to generate an in-frame splice variant of HTT (Fig 3E). No post-translational modifications or structural features have so far been discovered in this sequence.

### All 4 new shorter isoforms are expressed during neural differentiation

**Fig 4:**
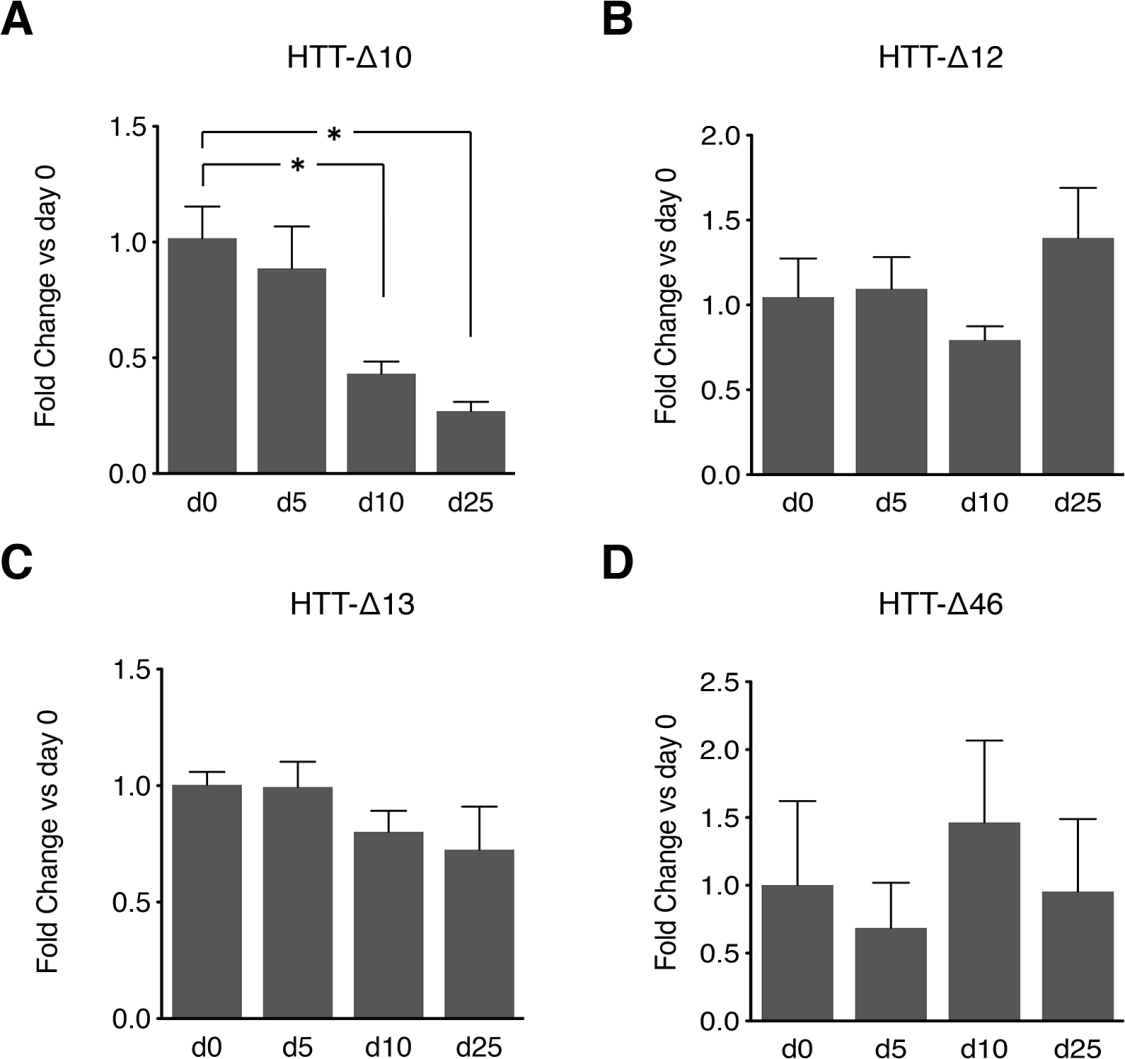
Time course of *HTT* isoform expression in hESCs differentiating to telencephalic neural fate. (A-D) RUES2 hESCs were differentiated to neural fate by blocking both branches of TGFβ signaling (default mechanism) as described in Materials and Methods. Values are normalized by GAPDH and displayed as fold change to day 0 values. Only the HTT-Δ10 isoform consistently decreases as the cells differentiate, while all three other isoforms maintain their expression levels unchanged. Error bars represent the standard error of the mean of 3 to 6 independent replicates. * p<0.05 vs d0.

In a recent study, embryonic stem cells were shown to express the highest number of splice isoforms, with diversity decreasing as the cells differentiate[26]. Because of the importance of HTT functions to neuronal maintenance, we asked whether any of the novel isoforms were enriched in neurons. We used inhibition of both branches of the TGFβ signaling pathway (“default”) to generate neuronal precursors (S2 Fig) and assessed isoform expression during the course of neuronal differentiation (Fig 4). While all 4 isoforms are expressed at all time-points analyzed, *HTT-Δ10* expression decreased during neural differentiation consistent with our results from *Xenopus* (S1 Fig). *HTT-Δ10* might therefore have a prominent role in pluripotent cells as well as in the induction of neural fate, but it might be excluded from mature neurons. The expression of the remaining isoforms was uniform over the time course.

### New hominid-specific exon: HTT-41b

**Fig 5:**
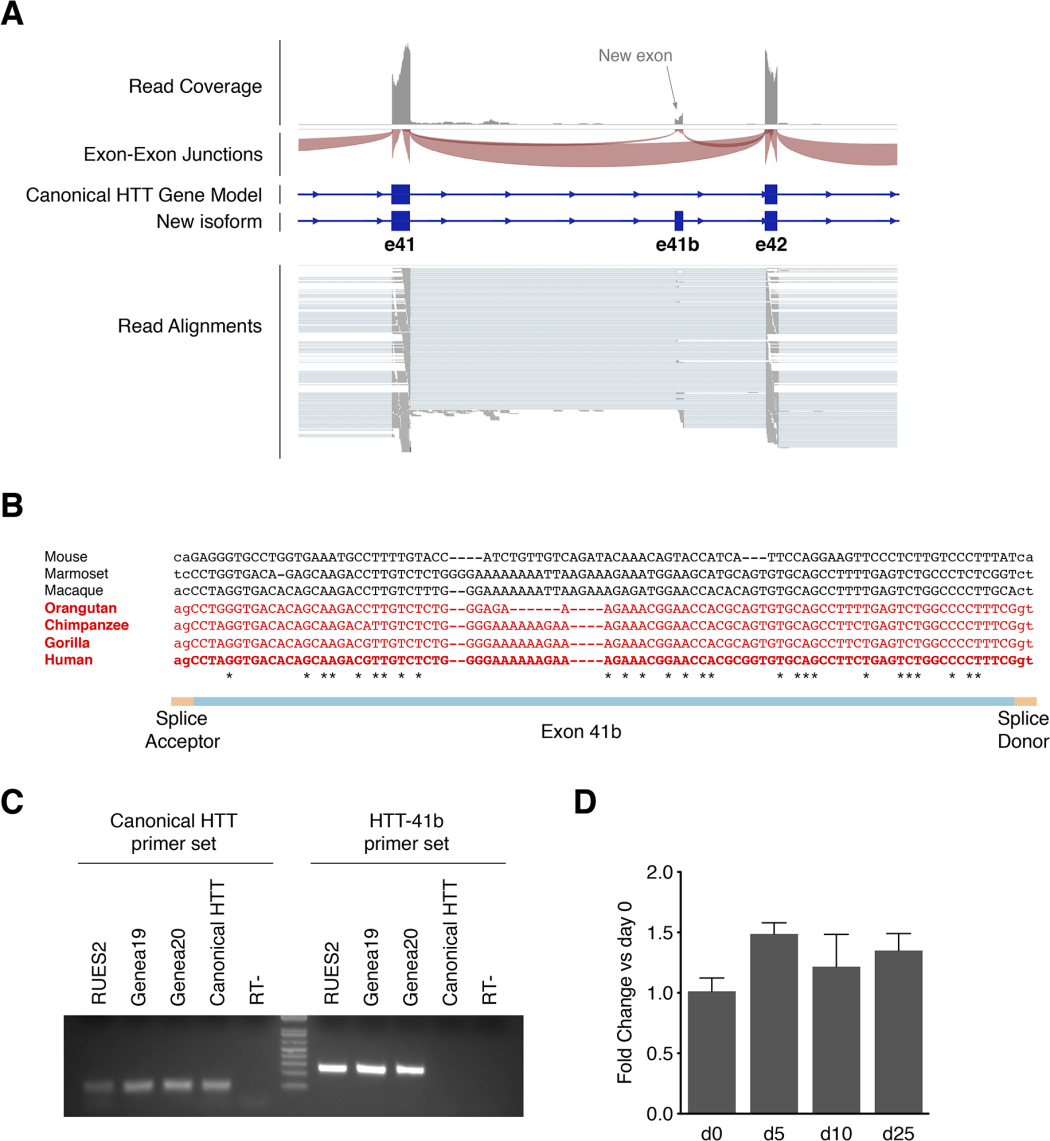
Identification of a novel HTT isoform incorporating a previously unreported hominid-specific exon. (A) RNAseq analysis showing the clear incorporation of a non-reported exon. (B) Alignment of the genomic sequences of exon 41b of mouse and primates, demonstrating the very recent acquisition of this exon along human evolution. Only hominidae family members (in red) have both splice donor and acceptor and maintain the frame. (C) RT-PCR with primers specific for HTT-41b isoform unmistakably detects expression of HTT-41b in hES cells, without amplifying the canonical HTT isoform. (D) Quantification of HTT-41b isoform expression through qRT-PCR at different time points upon neural differentiation of hES cells. Results are shown as mean+SEM of 3–6 independent replicates.

This isoform incorporates a previously unreported exon located between exons 41 and 42 (hence named 41b), which potentially adds 30 new amino acids to the canonical HTT protein (Fig 5A). Strikingly, both the coding frame and the splice acceptor-donor sites are exclusively present in Great Apes (orangutan, gorilla, chimpanzee) and humans, reflecting a very recent acquisition in the human evolutionary scale (Fig 5B). Indeed, the nucleotide sequence of the exon region suggests that it was acquired through the insertion of a new Alu element during evolution of the hominidae family. Thus, this discovery could explain at least some of the phenotypic differences observed between HD patients and mouse models. This HTT-41b isoform was validated by PCR in hESCs and its expression was stably maintained upon neural differentiation as determined by qPCR (Fig 5C and 5D). Importantly, the HTT-41b isoform was the only isoform clearly detected in RNA-seq samples of human adult brain cortex (data analyzed from GEO accession number GSE59612)[27], and its expression level was similar to what was detected in hESCs, ranging from 7.5–14% compared to the major HTT isoform. In fact, the HTT-41b isoform was clearly identified in all human adult tissues analyzed in the Illumina’s BodyMap 2.0 project (S3 Fig), suggesting that this isoform is ubiquitously expressed.

No structural domain has been detected in these 30 amino acids, but the addition of these extra amino acids could add new phosphorylation sites (S4 Fig), as well as generate conformational changes that may affect or modify its function. Such a hominid-specific isoform may help explain phenotypic differences between human patients and current mouse models of HD.

## Materials and Methods

### hESC culture and differentiation

RUES2 hESC line was originally derived in our laboratory[13,14] (NIH hESC-09-0013). GENEA019 and GENEA020 lines were obtained from Genea Biocells (Australia). All three hESC lines were maintained under pluripotency conditions in MEF-conditioned medium in presence of 20ng/ml bFGF. Medium was changed every day and cells were passaged once a week to avoid overcrowding as previously described[13]. RUES2 hESC were differentiated to neural lineage following previously described methods[28] with minor modifications. Briefly, embryoid bodies (EBs) were formed from ES cell colonies (in 20uM ROCK inhibitor Y27632, for the first two days) and treated with TGFβ signaling inhibitors (10uM SB-431542 and 0.2uM LDN-193189) every other day for 10 days. At day 10 EBs were dissociated and seeded on poly-ornithine laminin coated dishes at 0.25M cells/cm2, cultured for another 13 days in medium with recombinant BDNF (10ng/ml, R&D), Ascorbic Acid (20uM), and db-cAMP (Sigma, 1uM). Cultures were fixed or lysed in Trizol at day 0, 5, and 10 and 23. RNA was purified using RNAeasy columns and cDNA was synthesized with Superscript III and random hexamer primers. Neural differentiation protocol was validated using qPCR for PAX6 (f- ATC ATA ACT CCG CCC ATT CAC; r- GCA AAT AAC CTG CCT ATG CAA) and EMX2 (f- GGT TAA TAT GGT GCG TCC CTT; r- GAT ATC TGG GTC ATC GCT TCC) and expression was normalized to GAPDH (f- TCT CTT CCT CTT GTG CTC TTG; r- CAC TTT GTC AAG CTC ATT TCC TG). As validation of proper neural differentiation, at discrete time points along the neural differentiation protocol, EBs were fixed with 4% PFA, cryoprotected, sectioned at 12um and along with fixed adherent cultures (day23), stained for markers of pluripotency (OCT4: BD, mouse 1:100; NANOG: EMD, rabbit 1:100), neural plate (SOX1: R&D, goat 1:500; PAX6: BD, mouse, 1:200), and neurogenic neuroepithelium (SOX9: R&D, goat 1:500).

### High-throughput sequencing and analysis

High-throughput sequencing was performed at The Rockefeller University Genomics Core Resource Center, using an Illumina HiSeq2000 instrument. In order to obtain longest reads and increase the potential for reading through a splice site, 100bp, single-end reads were obtained. Raw data is publicly available on NCBI GEO (accession GSE66769). Sequencing reads in the sequencer output file were aligned to the reference genome (hg19) through TopHat2[29] and using UCSC hg19 annotations as a reference transcriptome. The total number of reads mapping to the HTT locus in each sample was calculated with the Samtools toolbox. Alternatively spliced HTT transcripts were visualized with the Integrative Genomics Viewer (IGV) software. Quantification of the expression of the splicing isoforms was performed by dividing the read coverage of the isoform-specific junction (or the average of two junctions, in the case of a novel exon) by the read coverage of the canonical isoform junctions.

### Isoform-specific PCR and qPCR

Total RNA was isolated using Trizol Reagent and RNeasy mini kits (QIAGEN), following manufacturer’s instructions. In-column DNAse treatment was performed during the purification to avoid any genomic DNA contamination (QIAGEN RNase-Free DNase Set). cDNA was generated using Transcriptor First Strand cDNA Synthesis Kit (Roche) and an oligo-(dT)_18_ primer, to ensure retrotranscription of polyadenylated transcripts. In order to amplify HTT splice isoforms specifically, we designed assays where one primer is common for both long and short isoforms, while the second primer specifically binds to the short isoform. This strategy was successful in amplifying short isoforms of HTT from cDNA templates prepared from hESC RNA. Non-specific amplification of the full-length isoform was assessed using a plasmid containing full-length HTT sequence. The amplicons obtained from the plasmid template were longer than those obtained from the cDNA templates and they were absent in cDNA lanes, showing minimal or no amplification. Both amplicons were sequenced for verification. The primers used for detecting splice isoforms are listed in Table 1.

**Table 1:** Primers used for detection of HTT splice isoforms.

Primer	PCR	qPCR
WTHtt-F:		GAGTATTGTGGAACTTATAGCTGG
WTHtt-R:		GCTGACATCCGATCTCGAT
ΔExon10-F:	GCAGCAGCAGGTCAAGGACA	TGTGGAACTTATAGGCAAAGTGC
ΔExon10-R:	CCTAAGAGCACTTTGCCTA	GCTGACATCCGATCTCGATT
ΔExon12-F:	CTGGTGGCCGAAGCCGTAGT	ACAGCTCCAGCCAGGTGTTA
ΔExon12-R:	TCGGTACCGTCTAACACCTG	CCATGGAAGAGTTCCTGAAG
ΔExon13-F:	TCTGCCACTGATGGGGATGA	AGACGGTACCGACAACCAGT
ΔExon13-R	ATGTGCCTGTTGAAGGGCTG	TGTTGAAGGGCTGTGGCTTC
ΔExon46-F:	GGGATCCATCTCAGCCAGTC	GGGATCCATCTCAGCCAGTC
ΔExon46-R:	TGGTGGAGAGACGAAACCTC	TGGTGGAGAGACGAAACCTC
Exon41-F:		CAGATACTGCTGCTTGTCAAC
Exon41-42-R:		ACAGACTGTGTCTTTTCGGG
Exon41b-F:		GTGACACAGCAAGACGTTG
Exon42-R:		CTCGGAGTCATGGAGGTTC

All PCR reactions were run for 40 cycles, 62°C annealing temperature using GoTaq PCR kit. All qPCRs were run for 45 cycles, 55°C annealing temperature using Lightcycler 480 Sybr green Master Mix (Roche). All results are expressed as the mean ± SEM. Statistical comparisons were made using 1-way analysis of variance (ANOVA), and multiple comparisons were made using Dunnett’s post-hoc test. A p-value less than 0.05 was considered statistically significant.

### Xenopus HTT isoform quantification

All procedures were approved by the Institutional Animal Care and Use Committee (IACUC) at the Rockefeller University (protocol number 14716-H). *Xenopus laevis* embryos were obtained by *in vitro* fertilization and staged according to Nieuwkoop and Faber (1967). The sequence of *X*.*laevis htt* cDNA was obtained from in-house RNAseq analysis of stage 10.5 embryos and aligned to *X*.*laevis* genome (XLaevis_JGIv7b) using the Tuxedo suite. Stage 35 embryos were anesthetized by MS-222 (tricaine methanesulfonate; Sigma-Aldrich), and dissected with a scalpel. The following primers were used for qPCR: To detect *htt-exon10i*, Exon9_F-ACT GGT TCT CTG GAG TTG CT and Exon10_R-TAC AGG GCT GCA GGT AGA AC; to detect *htt-∆exon10*, Exon9_F2-TCA GCG TTT GCA GAG ATG AG and Exon11_R-GAG GCA TCA GAC TTG CCT TC; to detect all htt, HTTall_F-CAC CGT GAT AGG CTC GTT CC, HTTall_R-GCT TTG GGT GCC GGC TCT TC. Odc was used as normalizer, odcF-GCC ATT GTG AAG ACT CTC TCC ATT C and odcR-TTC GGG TGA TTC CTT GCC AC.

## Supporting Information

S1 Fig Analysis of temporal and spatial expression of *htt* isoforms in *Xenopus laevis*. (A) RNAseq data of gastrula embryos revealed that exon10 is spliced out in more than 95% of the mRNAs. (B) Temporal expression of total *htt* (top), *htt-Δ10* (middle) and *htt-ex10i* (bottom) isoforms. Inclusion of exon 10 is enhanced starting at stage 28, while the *htt-Δ10* isoform expression is maintained constant. (C) Spatial expression of total *htt* (top), *htt-Δ10* (middle) and *htt-ex10i* (bottom) isoforms. The *htt-ex10i* isoform is enriched in head and dorsal sections, suggesting an increased expression of this isoform in the nervous system.
(TIF)

S2 Fig Validation of the telencephalic differentiation protocol. (A) qPCR quantification of PAX6 gene expression, showing the expected peak at the neural progenitors stage (d10). (B) qPCR quantification of EMX2, demonstrating a proper telencephalic fate acquisition at late time points. (C) Immunostaining of differentiating cultures for pluripotency markers (OCT4 and NANOG) and neural markers (SOX1 and PAX6), showing loss of pluripotency and gain of neural fate characteristics. All scale bars are 100 μm.
(TIF)

S3 Fig HTT-41b isoform is detected in all adult human tissues. Diagram quantifying all exon 41-41b-42 junctions detected in RNAseq samples from 16 different human adult tissues, from the Illumina’s BodyMap 2.0 project. HTT-41b is clearly detected in all 16 tissues, supporting the idea that this isoform is widely expressed.
(TIF)

S4 Fig Addition of exon 41b modifies serine/threonine phosphorylation predictions. Phosphorylation prediction of the region surrounding exon 41b in the major canonical HTT isoform (A) and with the incorporation of the novel exon 41b (B). Red asterisks indicate differences in phosphorylation site predictions.
(TIF)
