# Supplementary figures and images for: Discovery of Novel Isoforms of Huntingtin Reveals a New Hominid-Specific Exon
Source: PLoS One. 2015 May 26;10(5):e0127687. doi: 10.1371/journal.pone.0127687 (PMC4444280; doi:10.1371/journal.pone.0127687)

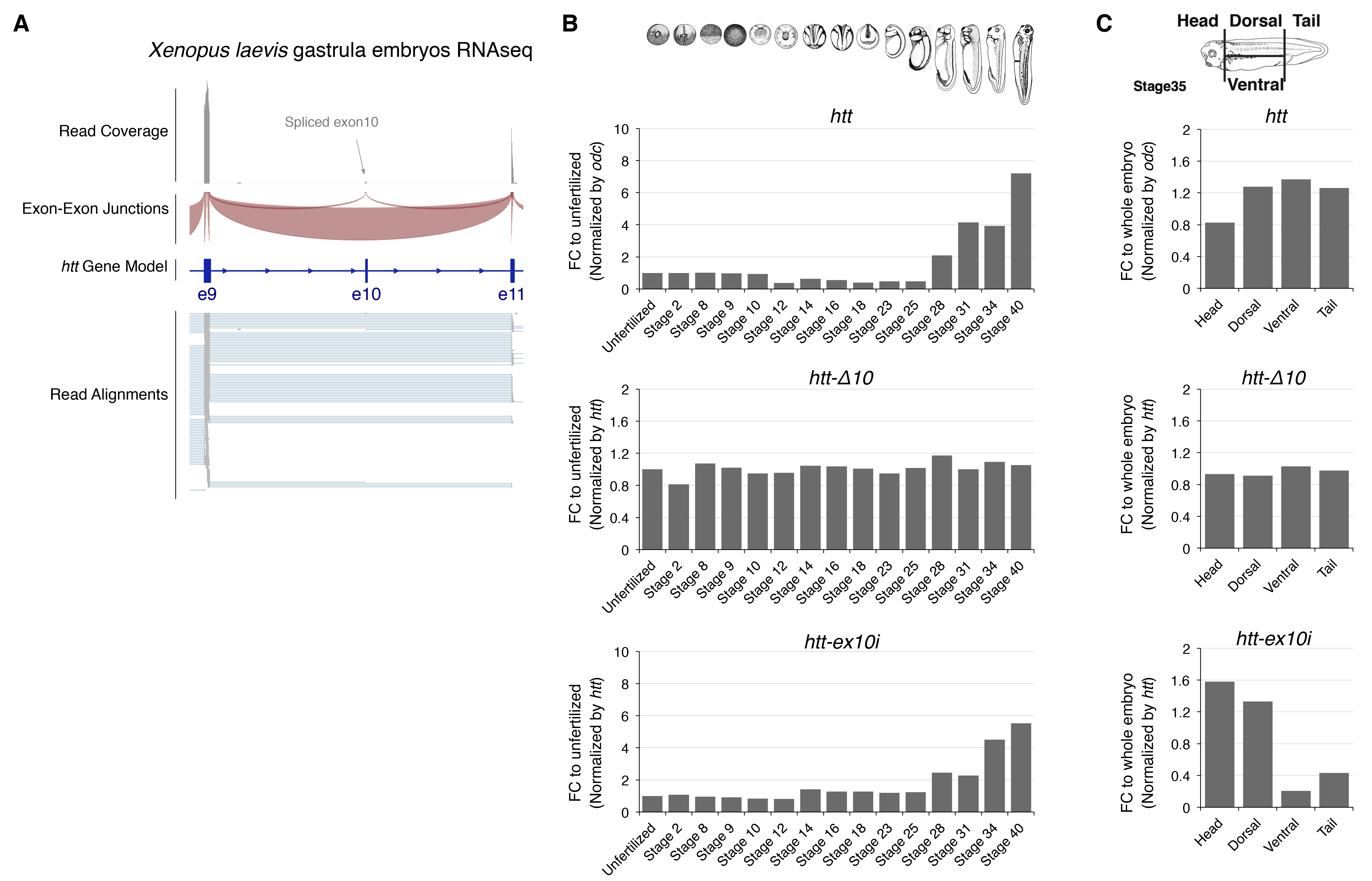

Supplement: S1 Fig — (A) RNAseq data of gastrula embryos revealed that exon10 is spliced out in more than 95% of the mRNAs. (B) Temporal expression of total htt (top), htt-Δ10 (middle) and htt-ex10i (bottom) isoforms. Inclusion of exon 10 is enhanced starting at stage 28, while the htt-Δ10 isoform expression is maintained constant. (C) Spatial expression of total htt (top), htt-Δ10 (middle) and htt-ex10i (bottom) isoforms. The htt-ex10i isoform is enriched in head and dorsal sections, suggesting an increased expression of this isoform in the nervous system. (TIF) [file pone.0127687.s001.tif]

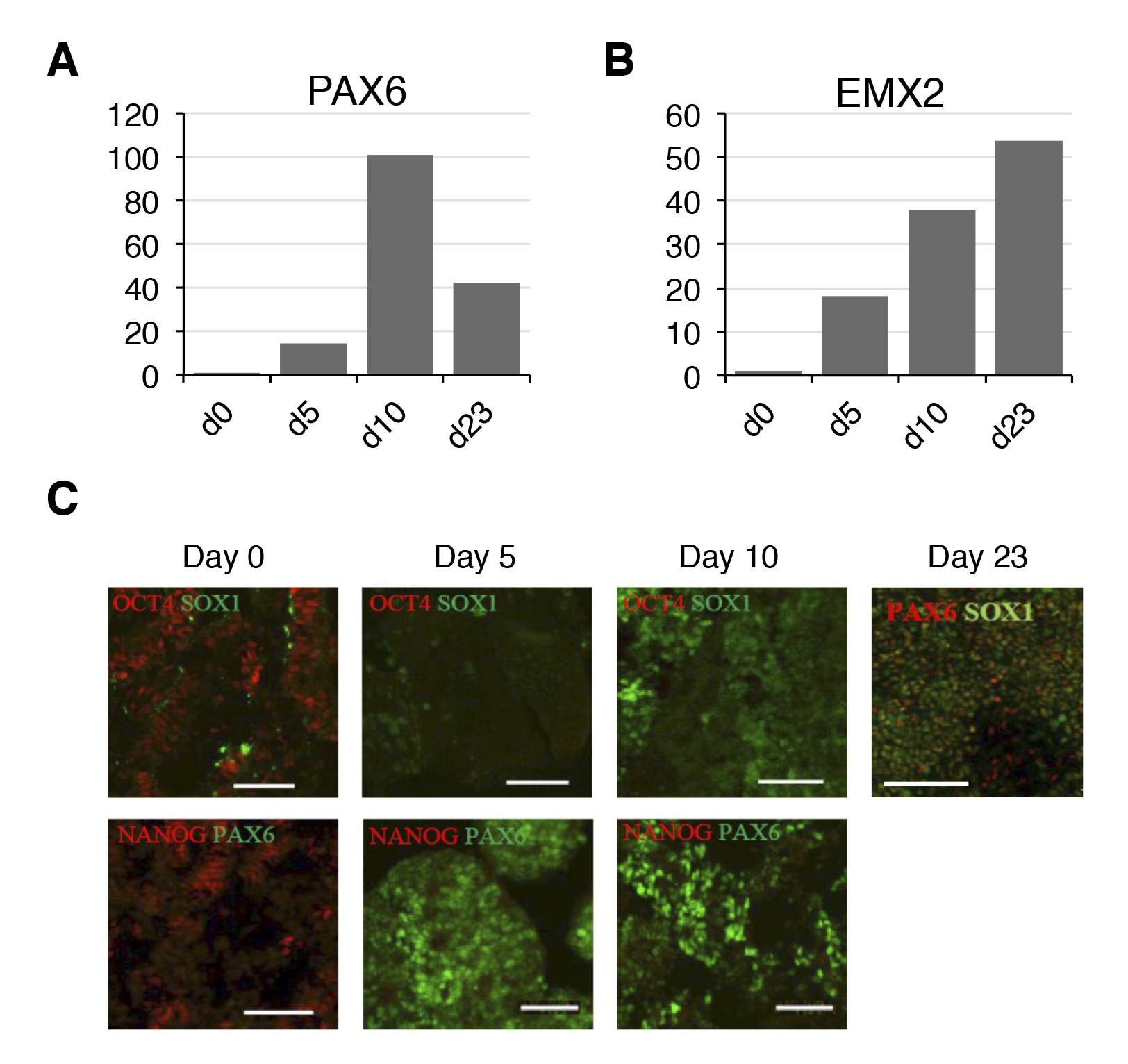

Supplement: S2 Fig — (A) qPCR quantification of PAX6 gene expression, showing the expected peak at the neural progenitors stage (d10). (B) qPCR quantification of EMX2, demonstrating a proper telencephalic fate acquisition at late time points. (C) Immunostaining of differentiating cultures for pluripotency markers (OCT4 and NANOG) and neural markers (SOX1 and PAX6), showing loss of pluripotency and gain of neural fate characteristics. All scale bars are 100 μm. (TIF) [file pone.0127687.s002.tif]

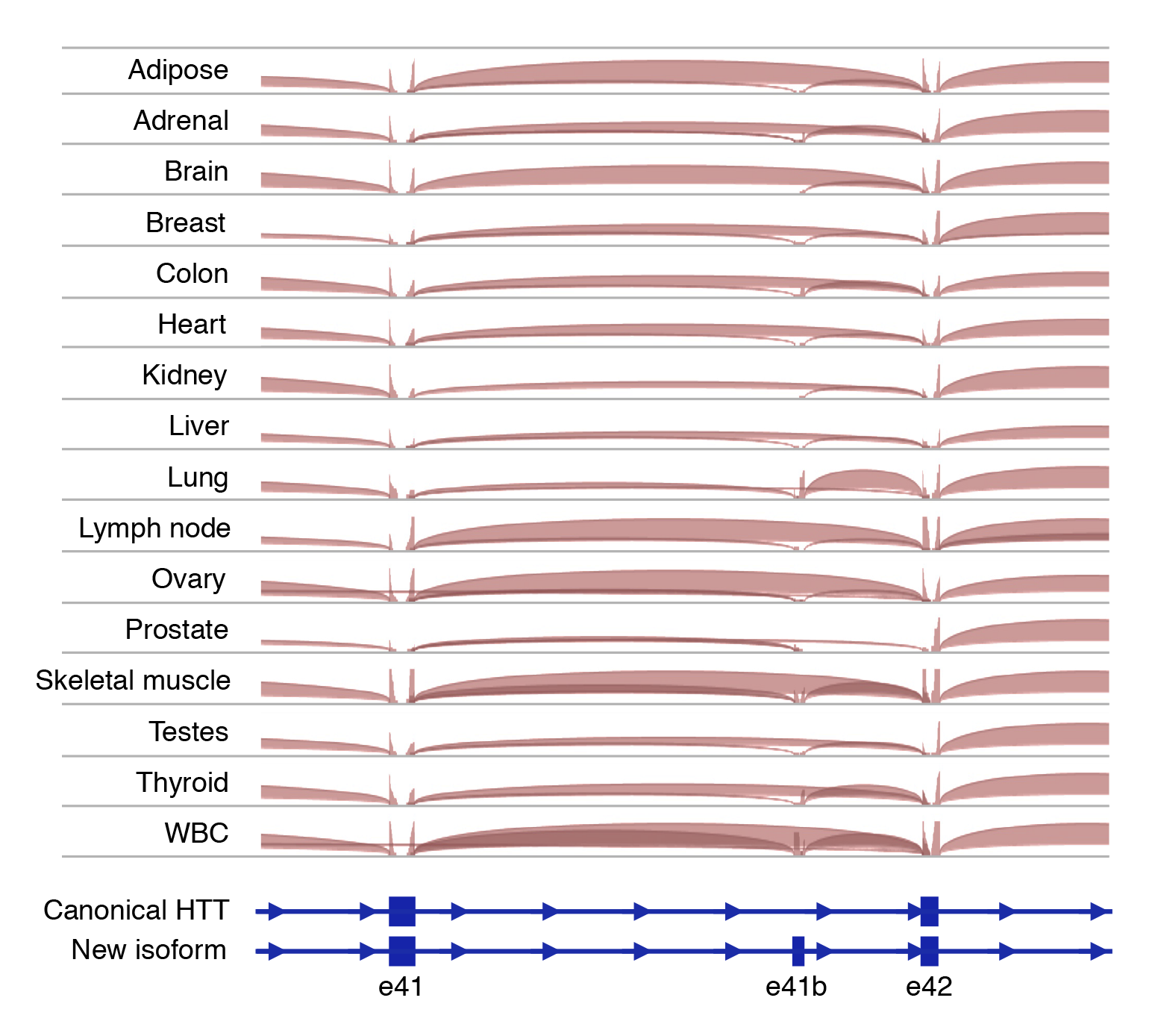

Supplement: S3 Fig — Diagram quantifying all exon 41-41b-42 junctions detected in RNAseq samples from 16 different human adult tissues, from the Illumina’s BodyMap 2.0 project. HTT-41b is clearly detected in all 16 tissues, supporting the idea that this isoform is widely expressed. (TIF) [file pone.0127687.s003.tif]

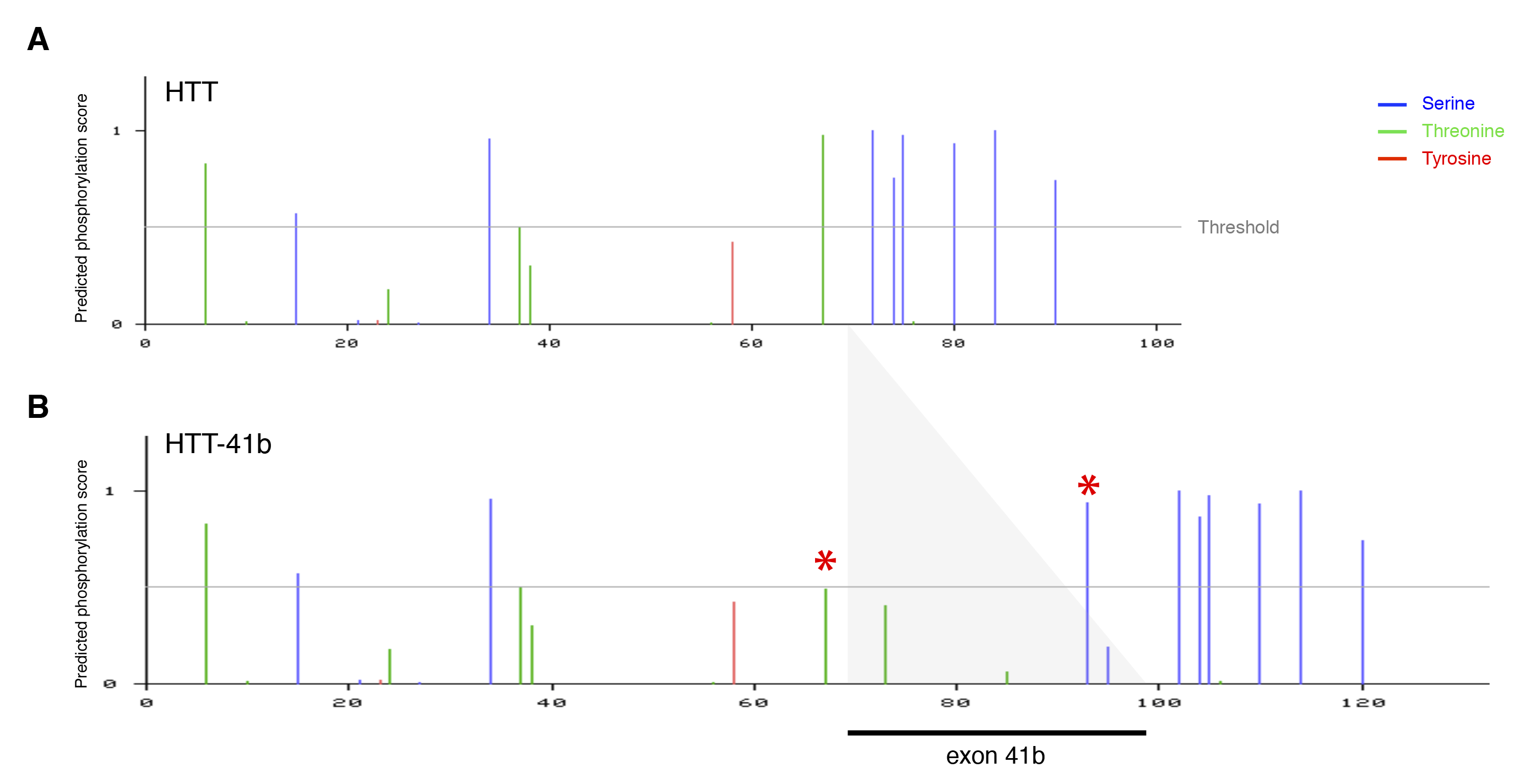

Supplement: S4 Fig — Phosphorylation prediction of the region surrounding exon 41b in the major canonical HTT isoform (A) and with the incorporation of the novel exon 41b (B). Red asterisks indicate differences in phosphorylation site predictions. (TIF) [file pone.0127687.s004.tif]
